# Highly sensitive and multiplexed quantification of mRNA splice variants by the direct ligation of DNA probes at the exon junction and universal PCR amplification†
†Electronic supplementary information (ESI) available. See DOI: 10.1039/c7sc00094d



**DOI:** 10.1039/c7sc00094d

**Published:** 2017-03-01

**Authors:** Honghong Wang, Hui Wang, Xinrui Duan, Yuanyuan Sun, Xiangdong Wang, Zhengping Li

**Affiliations:** a Key Laboratory of Analytical Chemistry for Life Science of Shaanxi Province , School of Chemistry and Chemical Engineering , Shaanxi Normal University , Xi’an 710062 , Shaanxi Province , P. R. China . Email: duanxr@snnu.edu.cn ; Email: lzpbd@snnu.edu.cn

## Abstract

Alternative messenger RNA (mRNA) splicing is a basic mechanism of gene regulation. In general, reverse transcription and polymerase based primer extension limit the sensitivity and selectivity of the current detection of mRNA splice variants, respectively. Here, we show that, using the ligation of two properly designed probes at the exon junction combined with universal PCR amplification, as little as a single copy of a mRNA splice variant per cell can be accurately determined, and the dynamic range covers six orders of magnitude. Three mRNA splice variants were measured from total RNA samples derived from different cell lines. Moreover, by encoding the ligation probes with different lengths, multiplexed mRNA splice variants can be simultaneously detected in one-tube PCR amplification using electrophoretic separation.

## Abstract 2

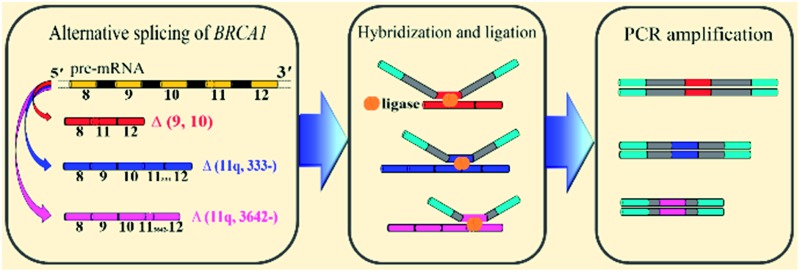
A highly sensitive and specific assay for detecting mRNA splice variants is developed based on ligation-dependent PCR.

## Introduction

Alternative pre-mRNA splicing is a centrally regulated process during gene expression in higher eukaryotes, in which particular exons from a single pre-mRNA can be spliced together in different arrangements to form distinct mature mRNA isoforms. Alternative splicing is a major source of the structural and functional diversity of proteins, which can explain how mammalian complexity arises from a surprisingly small complement of genes, and thus plays critical roles in various biological processes, including cell differentiation and development, homeostatic activities, and responses to extracellular stimuli. Aberrant variants in alternative splicing are implicated in a variety of diseases, including cystic fibrosis, Parkinsonism, spinal muscular atrophy, myotonic dystrophy, premature aging, and cancer.^1–3^ Genome-wide mapping of alternative splicing in the human transcriptome has been intensively studied using the sequencing of expressed sequence tags (ESTs)^4^ and high-density DNA microarrays.^5,6^ In particular, massively parallel (high throughput next generation) sequencing technologies have been recently applied to the surveying of alternative splicing complexity in the human transcriptome,^7–9^ and have provided unprecedentedly deep and comprehensive sequencing data for alternative splicing, making it possible to apply powerful statistical techniques for the discovery of the relationships between the functions, diseases, and alternative splicing variants.^10^ Based on deep sequencing technologies, it has been proven that more than 90% of human genes undergo alternative splicing.^9,10^ As the contribution of alternative splicing events to biological processes and disease diagnosis becomes increasingly recognized, sensitive and precise methods of quantifying the abundance of mRNA splice variants in biological and clinical samples have also become increasingly important, especially for the detection of selected splicing events in the disease-related genes that are routinely run in ordinary laboratories. In this respect, the high throughput technologies mentioned above are seriously limited by their laborious steps and the high cost of reagents and equipment, as well as the requirement of a large amount of the mRNA sample.

So far, the reverse transcription polymerase chain reaction (RT-PCR),^11^ real-time PCR in particular,^12,13^ has been most commonly used to quantitatively detect mRNA splice variants, and these can obtain a wide dynamic range of quantification and high sensitivity. However, these PCR-based methods have shown instinctive limitations. The PCR amplification of the specific splicing isoform is generally achieved by a boundary-spanning primer (BSP) hybridizing to the sequence encompassing the exon–exon junction. Mispriming due to a partial match between either the 5′ or 3′ end of the BSP and the other splicing isoform often results in erroneous amplification and thus makes isoform-specific detection challenging.^13^ In addition, the reverse transcription in the RT-PCR increases the cost and the operation steps. More recently, a variety of methods have been developed for the quantification of splice variants based on surface-enhanced Raman spectroscopy,^14,15^ microarrays,^16,17^ and matrix-assisted laser desorption/ionization time-of-flight mass spectrometry.^18^ Moreover, J. Zhu and J. A. Butz *et al.* have investigated digital polony exon profiling techniques^19,20^ and K. Lee *et al.* have developed quantitative imaging of a single mRNA splice variant in living cells.^21^ Although these newly developed methods have made great advances for the multiplexed analysis of mRNA splice variants, the requirements of sophisticated procedures and/or expensive instruments have restricted their widespread application in ordinary laboratories and clinical diagnosis.

Theoretically, the most direct and reliable method of discriminating the splicing isoforms is to ligate the two oligonucleotide probes at the exon–exon junction sites. The ligation reaction catalyzed by DNA ligase has shown high-specificity for discrimination between complementary and mismatched oligonucleotides compared to the primer extension reaction catalyzed by DNA polymerase.^22^ Nevertheless, the ligation efficiency and specificity of oligonucleotide probes are seriously challenging when using RNA as the template.^23^ We have solved these challenges using T4 RNA ligase 2 to improve the ligation specificity and by modifying one oligonucleotide probe with two ribonucleotides at its 3′-end^23,24^ to greatly improve the ligation efficiency,^24^ which makes it possible to develop a practical ligation-based method for the detection of mRNA splice variants. Based on these, in this work, we have newly developed a highly sensitive and multiplexed strategy for the quantification of mRNA splice variants by the direct ligation of oligonucleotide probes at the exon junction and universal PCR amplification. The proposed method has exhibited several advantages that can overcome the limitations associated with the existing methods as mentioned above. Firstly, the oligonucleotide probes can be simply designed to be complementary to the exon sequence in a specific splicing isoform adjacent to the exon–exon junction and can be efficiently ligated with high specificity, which can avoid the reverse transcription. Secondly, the two oligonucleotide probes have been designed with a universal sequence at their 3′ or 5′ end, and thus, the PCR amplification for any specific splicing isoform can be performed using an identical primer pair, permitting the efficient reduction of the sequence bias towards particular splicing isoforms. More importantly, by simply encoding the oligonucleotide probes with different lengths for different splicing isoforms, the proposed method can realize the multiplexed detection of mRNA splice variants in one tube PCR with common gel electrophoresis. Finally, the proposed method can be run routinely with simple operational procedures and commonly available instruments in ordinary laboratories.

## Results and discussion

### 1. The principle of the ligation-dependent PCR assay for the detection of mRNA splice variants

**Fig. 1:**
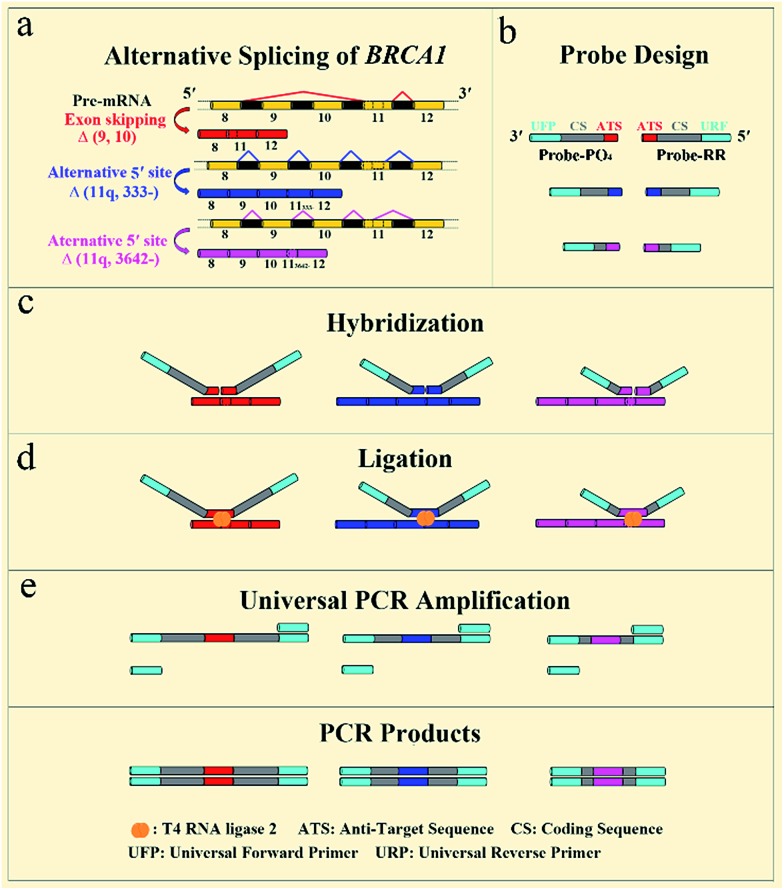
Schematic diagram of the direct ligation of DNA probes at the exon junction with universal PCR amplification for the detection of mRNA splice variants from the *BRCA1* gene.

The general outline of our strategy for the detection of mRNA splice variants is shown in Fig. 1. We chose three mRNA splice variants from breast cancer susceptibility gene 1 (*BRCA1*) pre-mRNA as model targets, including Δ (9, 10) (exon skipping, exon 9 and 10 deleted), alternative donor site Δ (11q, 333-) (alternative 5′ splice junction, the last 333 nt deleted from exon 11), and alternative donor site Δ (11q, 3642-) (alternative 5′ splice junction, the last 3642 nt deleted from exon 11) (Fig. 1a). *BRCA1* is a major gene that causes a genetic susceptibility to breast cancer and the profile of the alternative splicing of the *BRCA1* gene is associated with malignant transformation in breast cancer.^14^ Initially, ligation probe pairs including probe-RR and probe-PO_4_ were designed according to the sequences of different mRNA splice variants (Fig. 1b). Each probe contains an anti-target sequence (ATS), a coding sequence of different length (CS), and two universal sequences for PCR amplification (universal forward primer (UFP) and universal reverse primer (URP)). In addition, probe-RR was modified with two ribonucleotides at its 3′ terminus in order to effectively improve the ligation efficiency.^24^ Probe-PO_4_ was modified with a phosphate group at its 5′ terminus. The coding sequence of each ligation probe pair has a unique length that acts as a “size code” for its target. The universal sequences of ligation probe pairs have identical sequences at their 5′ and 3′ terminus, which permit simultaneous amplification in the one tube PCR with uniform PCR primers. All of the sequences of mRNA splice variants, the ligation probes, and the PCR primers are listed in Table S1 (see ESI†). In the presence of the target mRNA splice variants, probe-RR and probe-PO_4_, respectively, adjacently hybridize with the target mRNA splices (Fig. 1c), and are ligated through catalysis of T4 RNA ligase 2 (Fig. 1d). Then, the ligated probes with different lengths can be amplified using PCR by adding the universal forward primer and the reverse primer (Fig. 1e). SYBR Green I was utilized as the fluorescent dye for the real-time detection of the PCR produces. Moreover, the PCR products can also be analyzed using polyacrylamide gel electrophoresis (PAGE), and different mRNA splice variants can be detected simultaneously. The proposed assay is based on direct ligation of DNA probes at the exon junction. The ligation reaction can occur only when the corresponding target of the mRNA splice variant is present in the detected samples. On the contrary, the most commonly used RT-PCR methods generally employ a boundary-spanning primer (BSP) and opposing primer to amplify the cDNA of the target of the mRNA splice variant.^13^ As mentioned above, the mispriming can result in erroneous amplification even when the target is absent in the samples. The detailed specificity comparison is presented in the ESI (Fig. S1†), demonstrating the high specificity of direct ligation of the DNA probes and the nonspecific amplification arising from mispriming in RT-PCR.

### 2. The dynamic range and sensitivity of the mRNA splice variant assay

One gene can produce several mRNA splice variants, and the difference of the expression levels among mRNA splice variants could be very dramatic. So, a wide dynamic range and high sensitivity of the method are necessary criteria for quantitative detection of mRNA splice variants. To evaluate the dynamic range and sensitivity of our ligation-dependent PCR assay, the standard curves of three mRNA splice variants were established using synthetic mRNA sequences.

**Fig. 2:**
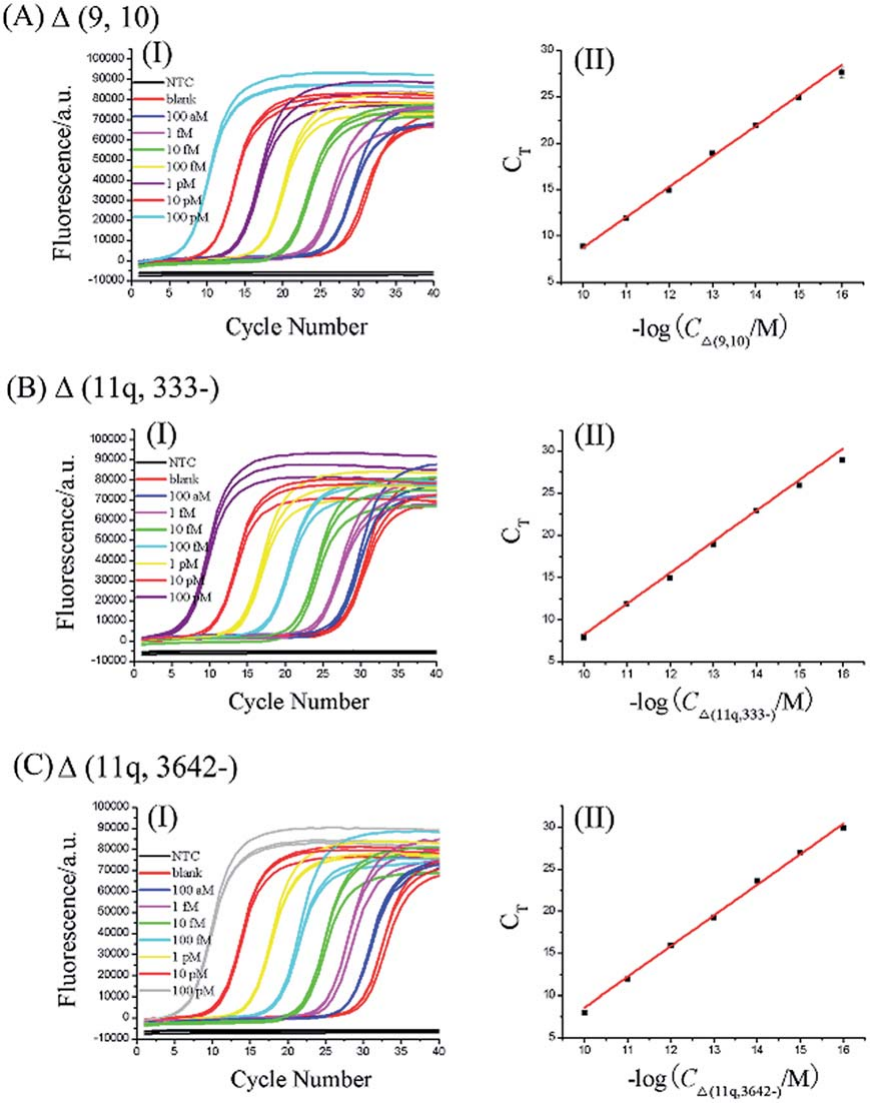
The dynamic range and sensitivity of the Δ (9, 10) (A), Δ (11q, 333-) (B), and Δ (11q, 3642-) (C) assays. In each figure panel of A–C, image (I) displays the real-time fluorescence curves of ligation-dependent PCRs produced by mRNA splice variants with different concentrations. From left to right, the concentration of the mRNA splice variant was successively 100 pM, 10 pM, 1 pM, 100 fM, 10 fM, 1 fM, and 100 aM, blank, and NTC. The blank was detected with the same procedures but without the mRNA splice variant. NTC was a control experiment where no ligation product was added into the PCR reaction. Each experiment was run in triplicate. (II) The linear relationship between the *C*_T_ values of the fluorescence curves and logarithm of the mRNA concentrations in image (I). Error bars indicate the standard deviation of three replicative tests.

According to the procedures described in the standard protocols (see ESI†), as shown in Fig. 2, as low as 100 aM of each mRNA splice variant can be accurately detected. Well-defined real-time fluorescence signals can be observed for Δ (9, 10), Δ (11q, 333-), and Δ (11q, 3642-) in concentrations ranging from 100 aM to 100 pM, with measurement of the fluorescence intensity produced by the ligation-dependent PCR. When the *C*_T_ values are plotted against the logarithm (log) of mRNA concentrations, as depicted in Fig. 2(II), there is an excellent linear relationship in the range from 100 aM to 100 pM. The correlation equations of Δ (9, 10), Δ (11q, 333-), and Δ (11q, 3642-) measurements were successively *C*_T_ = –24.1–3.28 log *C*_Δ (9, 10)_ (M) with the corresponding correlation coefficient *R* ^2^ = 0.9974, *C*_T_ = –28.5–3.67 log *C*_Δ (11q, 333-)_ (M) with the corresponding correlation coefficient *R* ^2^ = 0.9999, and *C*_T_ = –27.8–3.64 log *C*_Δ (11q, 3642-)_ (M) with the corresponding correlation coefficient *R* ^2^ = 0.9998, indicating that the proposed assay has a wide dynamic range over six orders of magnitude. Additionally, the similar slope of each calibration curve proves that the ligation-dependent PCR assay has good generality for different mRNA splice variants. Although the sensitivity of the proposed assay is quite high, small amounts of nonspecific amplification can be observed from the exponentially amplified signal of the blank, as shown in Fig. 2(I). The nonspecific amplification is generally inevitable^25^ and is a key issue to limit the sensitivity because the detection limit is determined by the minimum target which can produce the signal to be distinguished from the nonspecific blank signal. The nonspecific amplification should arise from the template-independent ligation of the DNA probes because (1) the nonspecific signal cannot be observed from the NTC, in which no ligation products are added. So the nonspecific signals should not be produced by the PCR primers. (2) As shown in Fig. S3 (see ESI†), the grayscale of the product band corresponding to the blank signal can be increased with increasing the amounts of the ligase (lane 2, lane 5 and lane 8). (3) T4 RNA ligase 2 has a small activity for the template-independent ligation according to its reagent instructions.

### 3. The specificity of the ligation-dependent PCR assay for the detection of mRNA splice variants

**Fig. 3:**
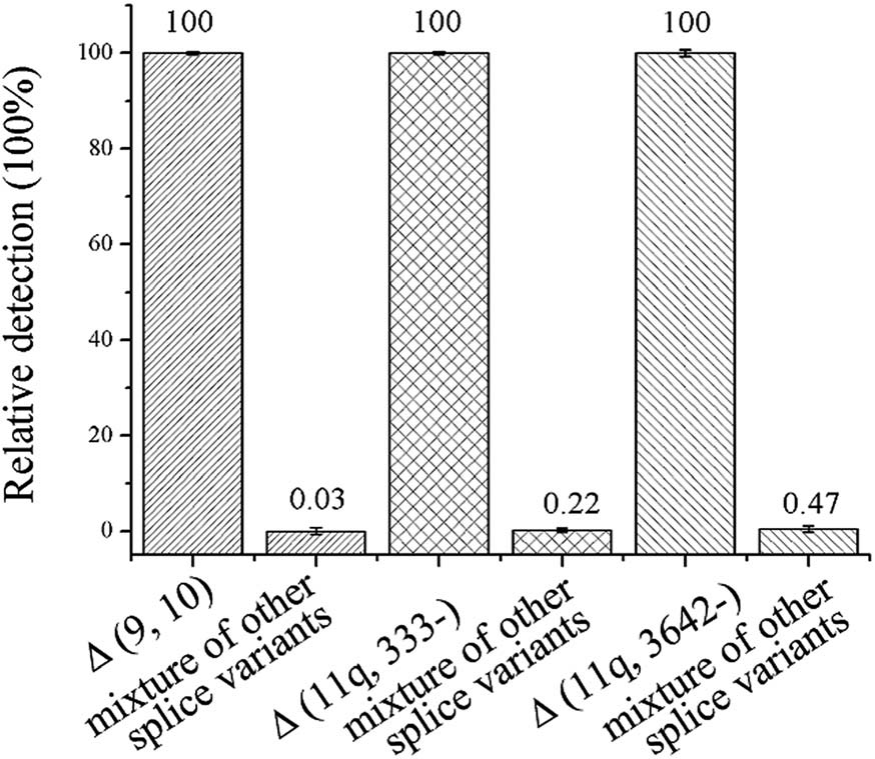
Δ (9, 10), Δ (11q, 333-), and Δ (11q, 3642-) were respectively detected with corresponding specific probes and the relative detection was defined as 100%. Meanwhile, the other two splice variants were mixed as the mixture of other splice variants which were also detected with the same specific probes. Each of the mRNA splice variants was 100 fM in concentration. Error bars indicate the standard deviation of three replicative tests.

The specificity of the method is key for distinguishing between the mRNA splice variants. To investigate the specificity of the proposed ligation-dependent PCR assay, Δ (9, 10), Δ (11q, 333-), and Δ (11q, 3642-) are detected respectively using the corresponding probe-RR and probe-PO_4_ in the ligation reaction. While one splice variant was detected with its specific probes, the other two splice isoforms were mixed as the interference sequence (mixture of other splice variants). The mixture was also detected with the same specific probes using the same procedures. The relative detection of the specific splice isoform was defined as 100% and the relative detection of the mixture of other targets was calculated by 1/2^Δ*C*_T_^ (Δ*C*_T_ = *C*_T, mixture of other splice variants_ – *C*_T, specific splice variants_). As shown in Fig. 3, the relative detection of the mixture of other splice variants by using the Δ (9, 10), Δ (11q, 333-), and Δ (11q, 3642-) specific probes was 0.03%, 0.22%, and 0.47%, respectively. The results showed that the nonspecific detection is less than 0.5%, therefore, the ligation-dependent PCR assay with high specificity is reliable enough to identify alternative splice variants of mRNA.

### 4. Multiplex analysis of the mRNA splice variants

**Fig. 4:**
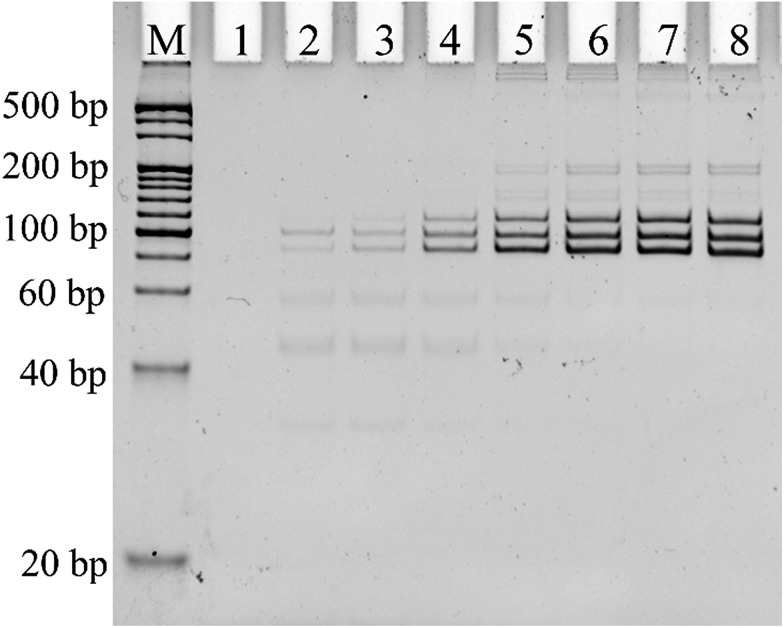
Non-denaturing polyacrylamide gel electrophoresis of multiplex analysis of mRNA splice variants. Lane M, double strand (ds) DNA markers; lane 1, NTC; lane 2, blank; and lanes 3 to 8, the mixture of mRNA splice variants (including Δ (11q, 3642-), Δ (11q, 333-), and Δ (9, 10)). The concentration of each mRNA variant is 1 fM (lane 3), 10 fM (lane 4), 100 fM (lane 5), 1 pM (lane 6), 10 pM (lane 7), and 100 pM (lane 8). The blank was characterized using the same procedures, but without the mRNA splice variant mixture. NTC was a control experiment where no ligation product was added into the PCR.

Alternative splicing can produce many different splicing isoforms from one pre-mRNA. Simultaneous detection of multiplexed isoforms in the one tube amplification reaction has great significance, especially for limited biological samples. Real-time PCR-based methods generally lack multiplexed isoform detection capability. Thus, we designed probe-RR and probe-PO_4_ with a unique length for each mRNA splice variant for the simultaneous detection of different mRNA splice variants. The PCR products of Δ (9, 10), Δ (11q, 333-), and Δ (11q, 3642-) were 114 bp, 97 bp, and 85 bp, respectively. 25 PCR cycles and 0.1 U μL^–1^ T4 RNA ligase 2 are found to be optimum for the multiplex assay (Fig. S2 and S3 in the ESI†). Under the optimum conditions, the PCR products of the ligation-dependent PCR were separated using PAGE in the presence of different concentrations of the mixture of the three mRNA splice variants. As shown in Fig. 4, the PCR products of the mixture of Δ (9, 10), Δ (11q, 333-) and Δ (11q, 3642-) show three defined bands corresponding to a 100 bp DNA marker (lane 3 to lane 8). With the concentration of the three mRNA splicing variants increasing, the number of ligation products acting as PCR templates gradually increased. More PCR templates can produce more amplification products by PCR amplification; the grayscale of the product band in the electrophoretogram became darker. The electrophoretogram in Fig. 4 shows that the three mRNA splice variants can be detected simultaneously to a concentration as low as 1 fM in one tube reaction with the ligation-dependent PCR assay. The combination of ligation probes with a “size code” and the capillary electrophoresis technique could achieve the simultaneous detection of nearly 40 different sequences,^26–28^ and, as far as we know, the average number of alternative mRNA splice variants of multi-exon genes is less than 20.^29^ Therefore, our method has the potential to analyze all the splice variants of pre-mRNA at the same time. We also used PAGE to investigate the specificity of the proposed ligation-dependent PCR assay (Fig. S4 in the ESI†). Similar to the results shown in Fig. 3 for real-time detection, the amplification product with the unique length corresponding to a mRNA splice variant can be generated only when the mRNA splice variant matched with the added probe-RR and probe-PO_4_. These results clearly indicate that the ligation-dependent PCR assay has a high specificity for distinguishing between the mRNA splice variants using PAGE as a detection tool.

### 5. Detection of the mRNA splice variants from total RNA samples

**Fig. 5:**
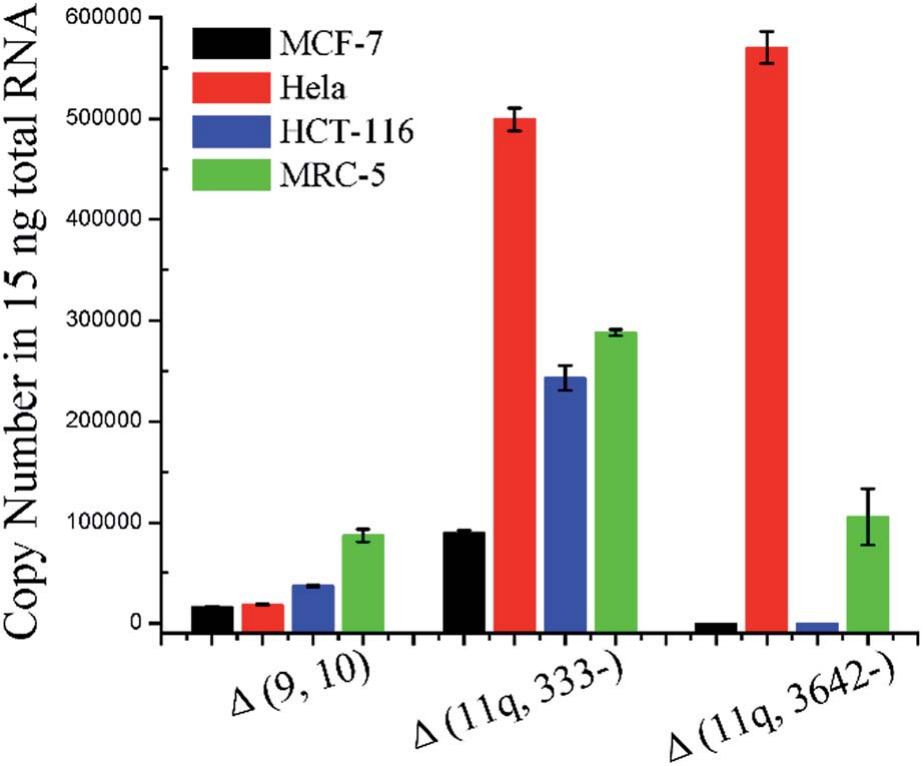
Quantification of *BRCA1* splicing events in 15 ng total RNA samples. The total RNA samples were extracted from MCF-7, Hela, HCT-116, and MRC-5 cells. Error bars indicate the standard deviation of three replicative tests.

In order to evaluate the practicality, the proposed ligation-dependent PCR assay has been applied to the detection of mRNA splice variants in a 15 ng total RNA sample derived from different cell lines, including a breast cancer cell line (MCF-7), a cervical cancer cell line (Hela), a colon cancer cell line (HCT-116), and a normal cell line (MRC-5). As shown in Fig. 5, the expression levels of the mRNA splice variants from different cell lines were different in the 15 ng total RNA sample. The quantification results presented in Fig. 5 reveal that the expression level of the Δ (9, 10) transcript was significantly higher in MRC-5 than in MCF-7, Hela, and HCT-116. The expression level of the Δ (9, 10) transcript in MCF-7 is consistent with previous reports.^21^ The transcript Δ (11q, 333-) has the highest expression level in Hela, a significantly lower expression level in HCT-116 and MRC-5, and the lowest expression level in MCF-7. The expression level of the Δ (11q, 3642-) transcript was clearly high in both Hela and MRC-5, and undetectable in MCF-7 and HCT-116. Since concentrations as low as 100 aM of each mRNA splice variant can be accurately detected, this is approximately 600 copies. Since samples of 15 ng total RNA are from approximately 1000 cells, we could expect the existence of 1000 copies of each mRNA splice variant in the 15 ng total RNA sample, even if one cell contains only a single copy of them. In other words, because our method is so sensitive, we could directly detect a single copy per cell of these three mRNA splice variants from a reasonable amount of total RNA sample. With the dynamic range of over six orders of magnitude in mind, our ligation based methods could quantitatively detect mRNA splice variants from the lowest copy number per cell (single copy) to the highest copy number per cell (10^5^ copies, the housekeeping gene beta-actin mRNA has a copy number near 10^5^ per cell and it is one of the most abundant mRNA in cells^30^) in a reasonable amount of the total RNA sample. Thus, our ligation-dependent PCR assay can be applied successfully to detect mRNA splice variants in total RNA samples from various cell lines.

## Conclusions

In conclusion, we have developed a highly selective and sensitive mRNA splice variant detection method without a reverse transcription reaction. By reasonably designing probe-RR and probe-PO_4_ according to the sequence of the target mRNA splice variant, the ligation-dependent mRNA splice variant assay exhibits excellent specificity and high sensitivity with a detection limit of 100 aM. The method was also extended to total RNA samples derived from different cell lines to demonstrate its feasibility as a quantitative diagnostic tool. Moreover, by encoding probe-RR and probe-PO_4_ with a unique length for each mRNA splice variant, the different mRNA splice variants can be detected simultaneously based on the length of the PCR products. Owing to the excellent specificity and high sensitivity, we believe that the proposed approach will find widespread applications in biomedical research and molecular diagnostics.
