# Supplementary material for: Highly sensitive and multiplexed quantification of mRNA splice variants by the direct ligation of DNA probes at the exon junction and universal PCR amplification
Source: Chem Sci. 2017 Mar 1;8(5):3635–40. doi: 10.1039/c7sc00094d (PMC5437374; doi:10.1039/c7sc00094d)
Supplement: Supplementary file 1 [file SC-008-C7SC00094D-s001.pdf]

Electronic Supplementary Material (ESI) for Chemical Science.
This journal is © The Royal Society of Chemistry 2017

Electronic Supporting Information

Highly Sensitive and Multiplexed Quantification of mRNA Splice Variants by Direct Ligation of DNA Probes at Exon Junction and Universal PCR Amplification

Honghong Wang,‡ Hui Wang,‡ Xinrui Duan,* Yuanyuan Sun, Xiangdong Wang and Zhengping Li*

Key Laboratory of Analytical Chemistry for Life Science of Shaanxi Province; School of Chemical and Chemical Engineering, Shaanxi Normal University, Xi'an 710062, Shaanxi Province, P. R. China.

Email: lzpbd@snnu.edu.cn; duanxr@snnu.edu.cn

Content List

1. Materials and apparatus

2. Preparation of total RNA extracts

3. Standard protocols of mRNA splice variants assay

4. Specificity comparison between RT-PCR and the direct ligation-based PCR

5. Optimization of PCR cycle number for multiplex mRNA splice variants assay

6. Optimization of T4 RNA ligase 2 dosage for multiplex mRNA splice variants assay

7. The specificity of mRNA splice variants assay using Non-denaturating PAGE

1. Materials and apparatus

T4 RNA ligase 2 (10 U/μL) and 10× T4 RNA ligase 2 reaction buffer (500 mM Tris-HCl, 20 mM $MgCl_2$, 10 mM DTT and 4 mM ATP, pH 7.5 @25°C) were purchased from New England Biolabs (USA). JumpStart^{TM} Taq DNA polymerase (2.5 U/μL) and 10× JumpStart^{TM} DNA polymerase reaction buffer (100 mmol/L Tris-HCl, 15 mmol/L $MgCl_2$, 500 mmol/L KCl and 0.01% (w/v) gelatin, pH 8.3 @25°C) were purchased from Sigma-Aldrich (USA). The MCF-7, Hela, HCT-116 and MRC-5 cell lines were purchased from the cell bank of Chinese Academy of Sciences (Shanghai, China). SYBR Green I (20 ng/μL stock solution in DMSO) was purchased from Xiamen Bio-Vision Biotechnology (Xiamen, China). Three synthetic mRNA splice variants of the BRCA1 using HPLC purified, ribonucleotide-modified DNA probes using HPLC purified, PO_4-modified DNA probes using HPLC purified, PCR primers using PAGE purified, dNTPs, RNase inhibitor (40 U/μL), RNase-free water, 20 bp DNA ladder and 6× nucleic acid sample loading buffer were purchased from TaKaRa Biotechnology Co. Ltd. (Dalian, China). Acrylamide, bisacrylamide, N, N, N′, N′-tetramethyl ethylenediamine (TEMED), ammonium persulfate (APS), trihydroxymethyl aminomethane (Tris), ethylene diamine tetraacetic acid (EDTA), boric acid and 4S Red Plus nucleic acid stain were obtained from Sangon Biotech Co. Ltd. (Shanghai, China). All the reagents were of analytical grade and were used as received without further purification. The sequences of the oligonucleotides were given in the **Table S1**.

The total RNA extracts were stored in Forma ultra-low temperature freezer (Thermo Scientific, USA). Temperature control device was 2720 PCR system (Applied Biosystems, USA). DYY-6D electrophoresis apparatus (Beijing Liuyi Biotechnology, China) and Gel DoxTM EZ Gel imaging system (BIO-RAD, USA) were used to perform electrophoresis separation and imaging analysis of PCR products. NanoDrop 2000 (Thermo Scientific, USA) was used to quantify the total RNA extracts. The real-time fluorescence measurements were performed with the StepOne Real-Time PCR system (Applied Biosystems, USA).

Table S1. The sequences of the oligonucleotides used in this work.

	ID	Sequences(5′-3′ direction)
mRNA	Δ (9, 10)	UCAAAAGACGUCUGUCUACAUUGAAUUG GCUGCUUGUGAAUUUCUGAGACGGAU
	Δ (11q, 333-)	AGAGCAAAGCAUGGAUUCAAACUUAGGU GAAGCAGCAUCUGGGUGUGAG
	Δ (11q, 3642-)	UGAGAGGCAUCCAGAAAAGUAUCAGGGU GAAGCAGCAUCUGGGTGUGAG
probe	Δ (9, 10) Probe-PO_4	PO_4- CAATTCAATGTAGACAGACGTCTTGTACCG CTTTCCTCTCTATGGGCAGTCGGTGAT
	Δ (9, 10) Probe-RR	CCATCTCATCCCTGCGTGTCTCCGACTCAG TTGTCTCAGAAAATTCACAAGCArGrC*
	Δ (11q, 333-) Probe-PO_4	PO_4- CCTGATACTTTTCTGGATGCCTCCTCTATG GGCAGTCGGTGAT
	Δ (11q, 3642-) Probe-PO_4	PO_4- CCTGATACTTTTCTGGATGCCTCCTCTATG GGCAGTCGGTGAT
	Δ (11q) Probe-RR	CCATCTCATCCCTGCGTGTCACACCCAGAT GCTGCTTCrArC*
primer	Forward Primer	ATCACCGACTGCCCATAGAG
	Reverse Primer	CCATCTCATCCCTGCGTGTC

* The letter “r” indicates “ribonucleotide”.

2. Preparation of total RNA extracts

The MCF-7, Hela, HCT-116 and MRC-5 cell lines were cultured in 6 mL DMEM Medium (GBICO, Cat. 12100-046) containing 10% (v/v) fetal calf serum (GBICO, Cat. 1600036), 1% $NaHCO_3$, 100 U/mL penicillin, 100 μg/mL streptomycin and 3 mmol/L L-glutamine. All of the cell lines were maintained at 37 °C under a humidified atmosphere containing 5% CO_2. Total RNA was extracted respectively from the MCF-7, Hela, HCT-116 and MRC-5 cell lines using a TRIzol® Reagent (Invitrogen, USA) according to the manufacture′s protocol.

3. Standard protocols of mRNA splice variants assay

(1) Standard protocols for mRNA splice variant assay by monitoring real-time fluorescence intensity:

1 μL mRNA splice variant or 1 μL total RNA extracts were added to the mixture containing 1 μL 200 nmol/L Probe-PO_4, 1 μL 200 nmol/L Probe-RR, 0.2 μL 40 U/μL RNase inhibitor, 0.6 μL 10× T4 RNA ligase 2 reaction buffer and 2.2 μL RNase-free water with final volume 6.0 μL. The mixture was heated at 65 °C for 2 min and at 37 °C for 5 min. Then 0.4 μL 10× T4 RNA ligase 2 reaction buffer, 0.1 μL 10 U/μL T4 RNA ligase 2 and 3.5 μL RNase-free water were added to the mixture, and the reaction mixture was incubated at 37 °C for 30 min to complete the ligation reaction.

2 μL ligation product was added to 8 μL PCR reaction mixture containing 0.2 μL 2.5 U/μL JumpStart™ DNA polymerase, 1.0 μL 10× JumpStart™ DNA polymerase reaction buffer, 1.0 μL 2.5 mmol/L dNTPs, 0.2 μL 10 μmol/L Forward Primer, 0.2 μL 10 μmol/L Reverse Primer, 0.2 μL 20 ng/μL SYBR Green I, and 5.2 μL RNase-free water with final volume 10 μL. PCR reaction were carried out with StepOne Real-Time PCR system to monitor real-time fluorescence intensity by using hot start of 94 °C for 2 min, followed by 40 cycles of 94 °C for 20 s, 60 °C for 30 s, and 72 °C for 20 s.

(2) Standard protocol for multiplex mRNA splice variants assay:

1 μL target mixture include different mRNA splice variants was added to the mixture containing 1 μL Probe-PO_4 mixture (the concentration of Probe-PO_4 corresponding each mRNA splice variant was 200 nmol/L), 1 μL Probe-RR mixture (the concentration of Probe-RR corresponding each mRNA splice variant was 200 nmol/L), 0.2 μL 40 U/μL RNase inhibitor, 0.6 μL 10× T4 RNA ligase 2 reaction buffer and 2.2 μL RNase-free water with final volume 6.0 μL. The mixture was heated at 65 °C for 2 min and at 37 °C for 5 min. Then 0.4 μL 10× T4 RNA ligase 2 reaction buffer, 0.1 μL 10 U/μL T4 RNA ligase 2 and 3.5 μL RNase-free water were added to the mixture, and the reaction mixture was incubated at 37 °C for 30 min to complete the ligation reaction.

2 μL ligation product was added to 8 μL PCR reaction mixture containing 0.2 μL 2.5 U/μL JumpStart™ DNA polymerase, 1.0 μL 10× JumpStart™ DNA polymerase reaction buffer, 1.0 μL 2.5 mmol/L dNTPs, 0.2 μL 10 μmol/L Forward Primer, 0.2 μL 10 μmol/L Reverse Primer, and 5.4

μL RNase-free water with final volume 10 μL. PCR reaction were carried out with 2720 PCR system by using hot start of 94 °C for 2 min, followed by 25 cycles of 94 °C for 20 s, 60 °C for 30 s, and 72 °C for 20 s. Different mRNA splice variants were detected simultaneously by separating amplification products using non-denaturing polyacrylamide gel electrophoresis. The gel was stained by 4S Red Plus Nucleic Acid Stain and visualized using a Gel Doc EZ Imager (Bio-Rad, USA).

4. Specificity comparison between RT-PCR and the direct ligation-based PCR

As shown as in Fig. S1 (A), there is a simplified and common model of alternative splicing of mRNA. The pre-mRNA that is composed of exon a, b, and c, which can be spliced into two mRNA splice variants: ac isoform and abc isoform. For detecting ac isoform with RT-PCR, a boundary-spanning primer (BSP, red arrow) and an opposing primer (black arrow) are generally designed as illuminated in Fig. S1 (B). The BSP can hybridize to the sequence encompassing the exon a^*-exon c^* in the cDNA and then extended along the cDNA with catalysis of DNA polymerase. The opposing primers will hybridize to the BSP-extended products and then extend along them catalyzed by DNA polymerase. And thus the BSP-extended products can be exponentially amplified by PCR. However, just as demonstrated by J. P. Brosseau et al.,[1] achieving isoform-specific amplification using BSP is challenging. The 3′ and 5′-end of the BSP perfectly match a part of the sequence in exon a^* and exon c^* in the cDNA of abc isoform, respectively. The perfect matching can result in a partial extension of BSP due to the mispriming and then the mispriming-extended products can also be exponentially amplified during PCR to produce false-positive signals. On the contrary, as demonstrated in Fig. S1 (C), for detecting ac isoform with the direct ligation-based PCR, the two DNA probes are respectively complementary to exon a and exon c. In the presence of ac isoform, the two DNA probes can adjacently hybridize to the isoform and are subsequently ligated with catalysis of ligase. The ligated products are then amplified by PCR. In the absence of ac isoform, the two probes can respectively hybridize to the exon a and exon c in the abc isoform, which are separated by exon b with a long distance. In addition, the DNA probes directly hybridize to RNA, and the ligase and DNA polymerase have not reverse transcriptase activity. So the probes cannot extend along the abc isoform. Therefore, the two DNA probes cannot be ligated. Namely, the ligation reaction can occur only when the

target of specific isoform is present in the detected samples, indicating the high specificity of the direct ligation-based PCR.

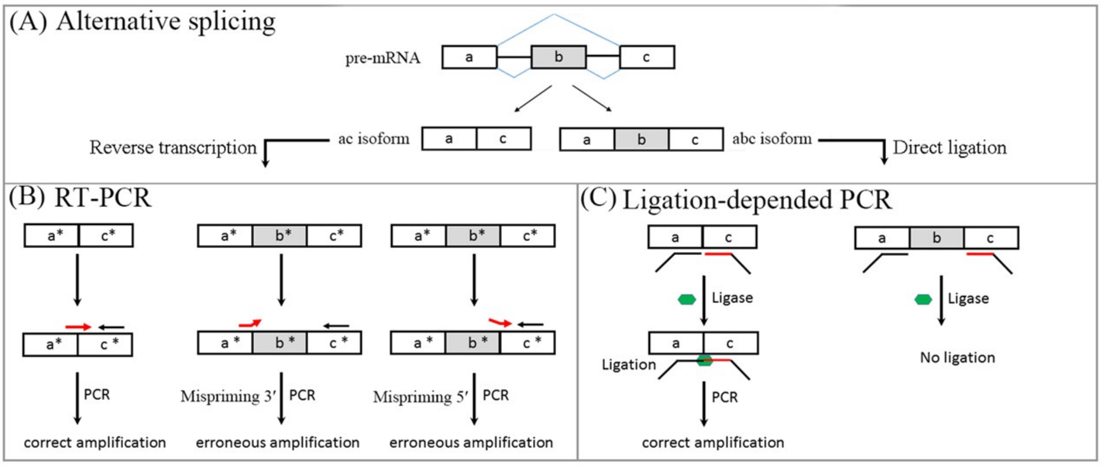

Fig. S1 (A) The model of alternative splicing of mRNA; (B)The principle of detection of mRNA splice variant by RT-PCR and mispriming-induced nonspecific amplification, a^*, b^*, c^* represent the corresponding cDNA after reverse transcription; (C) The principle of detection of mRNA splice variant by the direct ligation-based PCR.

5. Optimization of PCR cycle number for multiplex mRNA splice variants assay

PCR cycle numbers determine how much the PCR amplification products, and the electrophoresis detection need enough PCR amplification products to accomplish imaging analysis. So the PCR cycle number was an important parameter for ligation-depended PCR assay to detect different mRNA splice variants simultaneously. We chose three synthetic Δ (11q, 3642-), Δ (11q, 333-), and Δ (9, 10) as the model to optimize PCR cycle number for the multiplexed analysis of mRNA splice variants. The optimal PCR cycle number was investigated by detection the mixture including Δ (11q, 3642-), Δ (11q, 333-) and Δ (9, 10) at 0, 1 fM, 10 fM and 100 fM, respectively. After 23, 25 and 30 cycles, PCR amplification products were characterized by gel electrophoresis. As shown in Fig. S2, with the increasing the PCR cycle numbers, the grayscale of the products band from the mRNA splice variants mixture of the same concentration in the electrophoretogram became deeper. When PCR underwent 23 cycles, PCR amplification product bands of 0 and 1 fM mRNA splice variants (lane 2 and lane 3) cannot be seen, and this is due to less PCR cycle numbers produce less PCR amplification products. When PCR underwent 25 cycles, PCR amplification product bands of 0 and 1 fM mRNA splice variants (lane 6 and lane 7) can be seen,

and the grayscale of the band from 1 fM the mRNA splice variants mixture was deeper than 0 fM the mRNA splice variants mixture. When PCR underwent 30 cycles, as the same as 25 cycles, 1 fM the mRNA splice variants mixture can be detected distinctly. Taking into consideration of both reaction time and detection limit, 25 cycles was selected as the optimum PCR cycle for multiplex alternative splicing assay.

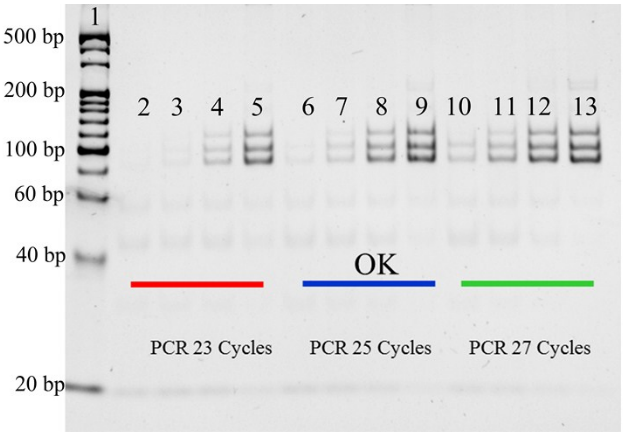

Fig. S2 Non-denaturating PAGE analysis of the amplification products of different thermal cycle number of ligation-depended PCR assay for Δ (11q, 3642-), Δ (11q, 333-) and Δ (9, 10). Lane 1, double strand (ds) DNA markers (20 bp DNA Ladder). Thermal cycle number of PCR is 23 cycles (lane 2 (0), lane 3 (1 fM), lane 4 (10 fM), lane 5 (100 fM)); 25 cycles (lane 6 (0), lane 7 (1 fM), lane 8 (10 fM), lane 9 (100 fM)); 27 cycles (lane 10 (0), lane 11 (1 fM), lane 12 (10 fM), lane 13 (100 fM)). Δ (11q, 3642-) products shows a defined band corresponding to 85 bp, Δ (11q, 333-) products shows a defined band corresponding to 97 bp, Δ (9, 10) products shows a defined band corresponding to 114 bp.

6. Optimization of T4 RNA ligase 2 dosage for multiplex mRNA splice variants assay

Ligase dosage not only affect the efficiency of ligation reaction but also the specificity of the ligation reaction. Therefore, T4 RNA ligase 2 dosage was investigated by testing Δ (11q, 3642-), Δ (11q, 333-), and Δ (9, 10) at 0, 1 fM, and 10 fM, respectively with the ligation-depended PCR assay. 0.05 U/μL, 0.1 U/μL, and 0.2 U/μL T4 RNA ligase 2 were investigated, as shown in Fig. S3. When T4 RNA ligase 2 dosage was 0.05 U/μL, 10 fM mRNA splice variants was detectable, and the bands of non-specific amplification products(lane 2) and amplification products of 1 fM mRNA splice variants (lane 3) can not seen. This is due to ligase dosage was too low, nonspecific

connections decreased, while specific connection between the probes and mRNA splice variants also decreased. Thus, the amount of the PCR amplified template was reduced, which did not produce enough amplification products to eletrophoresis separation. When T4 RNA ligase 2 dosage was 0.1 U/μL, the bands of non-specific amplification products(lane 5) and amplification products of 1 fM mRNA splice variants (lane 6) can be seen, and the bands grayscale of lane 6 were deeper than lane 5. That is say that 1 fM mRNA splice variants was detectable using 0.1 U/μL T4 RNA ligase 2. When T4 RNA ligase 2 dosage was 0.2 U/μL, the bands of non-specific amplification products(lane 8) and amplification products of 1 fM mRNA splice variants (lane 9) can also be seen, but the bands grayscale was different to distinguish between lane 8 and lane 9. Therefore, 0.1 U/μL was selected as the optimum T4 RNA ligase 2 dosage for multiplex alternative splicing assay.

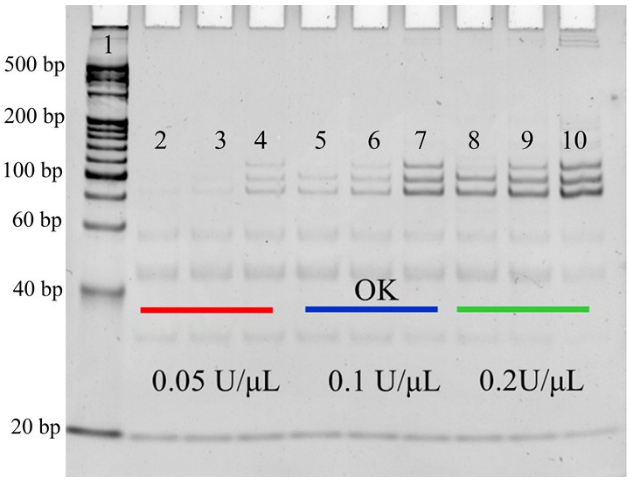

Fig. S3 The effect of T4 RNA ligase 2 dosage on the mRNA splice variants assay. Lane 1, double strand (ds) DNA markers (20 bp DNA Ladder). The concentration of ligase is 0.05 U/μL (lane 2 (0), lane 3 (1 fM), lane 4 (10 fM)); 0.1 U/μL (lane 5 (0), lane 6 (1 fM), lane 7 (10 fM)); 0.2 U/μL (lane 8 (0), lane 9 (1 fM), lane 10 (10 fM)). Δ (11q, 3642-) products shows a defined band corresponding to 85 bp, Δ (11q, 333-) products shows a defined band corresponding to 97 bp, Δ (9, 10) products shows a defined band corresponding to 114 bp.

7. The specificity of mRNA splice variants assay using non-denaturating PAGE

In order to further testify the specificty of the proposed ligation-depended PCR assay, the ampification products of multiplex mRNA splice variants are characterized by PAGE. We chose

the mixture including 100 fM Δ (11q, 3642-), 100 fM Δ (11q, 333-), and 100 fM Δ (9, 10) as target client. As shown in Fig. S4, when Δ (11q, 3642-) probes were added to ligation reaction, only Δ (11q, 3642-) products shows a defined band corresponding to 85 bp (lane 3). When Δ (11q, 333-) probes were added to ligation reaction, only Δ (11q, 333-) products shows a defined band corresponding to 97 bp (lane 4). When Δ (9, 10) probes were added to ligation reaction, only Δ (9, 10) products shows a defined band corresponding to 114 bp (lane 5). When Δ (11q, 3642-) probes and Δ (11q, 333-) probes were added to ligation reaction, only Δ (11q, 3642-) and Δ (11q, 333-) products shows two defined bands corresponding to 85 bp and 97 bp (lane 6). When Δ (11q, 3642-) probes and Δ (9, 10) probes were added to ligation reaction, only Δ (11q, 3642-) and Δ (9, 10) products shows two defined bands corresponding to 85 bp and 114 bp (lane 7). When Δ (11q, 333-) probes and Δ (9, 10) probes were added to ligation reaction, only Δ (11q, 333) and Δ (9, 10) products shows two defined bands corresponding to 97 bp and 114 bp (lane 8). When Δ (11q, 3642-) probes, Δ (11q, 333-) probes, and Δ (9, 10) probes were added to ligation reaction, Δ (11q, 3642-), Δ (11q, 333-), and Δ (9, 10) products shows three defined bands corresponding to 85 bp, 97 bp, and 114 bp (lane 9). In a word, amplification product of corresponding length of the mRNA splice variants could be generated only when corresponding target probes were added to the ligation reaction in the presence of three mRNA splice variants. The results indicate that ligation-depended PCR assay possess good specificity to distinguish different mRNA splice variants.

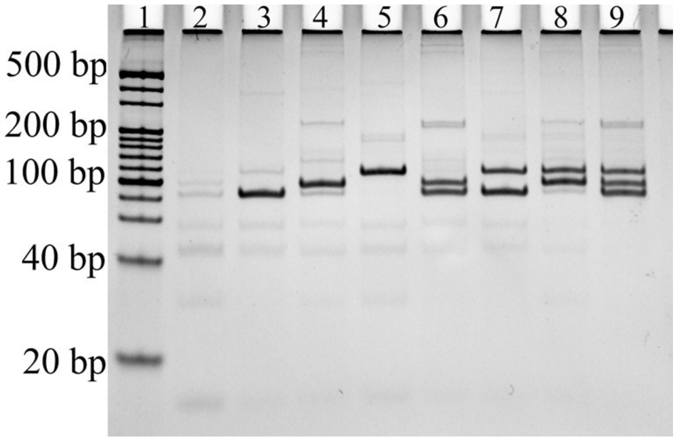

Fig. S4 The specificity of mRNA splice variants assay using specific DNA probes or different combinations of specific DNA probes in the ligation reaction. Each mRNA splice including Δ (11q, 3642-), Δ (11q, 333-), and Δ (9, 10) was 100 fM. Lane 1, double strand DNA markers; lane 2, blank; lane 3, Δ (11q, 3642-) probes; lane 4, Δ (11q, 333-) probes; lane 5, Δ (9, 10) probes; lane

6, Δ (11q, 3642-) probes and Δ (11q, 333-) probes; lane 7, Δ (11q, 3642-) probes and Δ (9, 10) probes; lane 8, Δ (11q, 333-) probes and Δ (9, 10) probes; lane 9, Δ (11q, 3642-) probes, Δ (11q, 333-) probes, and Δ (9, 10) probes. Δ (11q, 3642-) products shows a defined band corresponding to 85 bp, Δ (11q, 333-) products shows a defined band corresponding to 97 bp, Δ (9, 10) products shows a defined band corresponding to 114 bp.

Reference

1 J.-P. Brosseau, J.-F. Lucier, E. Lapointe, M. Durand, D. Gendron, J. Gervais-Bird, K. Tremblay, J.-P. Perreault and S. A. Elela, RNA, 2010, 16, 442-449.
